# Performance assessment in brain-computer interface-based augmentative and alternative communication

**DOI:** 10.1186/1475-925X-12-43

**Published:** 2013-05-16

**Authors:** David E Thompson, Stefanie Blain-Moraes, Jane E Huggins

**Affiliations:** 1 Department of Biomedical Engineering, University of Michigan, Ann Arbor, MI, USA; 2 Department of Physical Medicine and Rehabilitation, University of Michigan, Ann Arbor, MI, USA

**Keywords:** Brain-computer interface, Augmentative and alternative communication, Outcome measures, Information transfer rate

## Abstract

A large number of incommensurable metrics are currently used to report the performance of brain-computer interfaces (BCI) used for augmentative and alterative communication (AAC). The lack of standard metrics precludes the comparison of different BCI-based AAC systems, hindering rapid growth and development of this technology. This paper presents a review of the metrics that have been used to report performance of BCIs used for AAC from January 2005 to January 2012. We distinguish between Level 1 metrics used to report performance at the output of the BCI Control Module, which translates brain signals into logical control output, and Level 2 metrics at the Selection Enhancement Module, which translates logical control to semantic control. We recommend that: (1) the commensurate metrics Mutual Information or Information Transfer Rate (ITR) be used to report Level 1 BCI performance, as these metrics represent information throughput, which is of interest in BCIs for AAC; 2) the BCI-Utility metric be used to report Level 2 BCI performance, as it is capable of handling all current methods of improving BCI performance; (3) these metrics should be supplemented by information specific to each unique BCI configuration; and (4) studies involving Selection Enhancement Modules should report performance at both Level 1 and Level 2 in the BCI system. Following these recommendations will enable efficient comparison between both BCI Control and Selection Enhancement Modules, accelerating research and development of BCI-based AAC systems.

## Introduction

**Figure 1:**
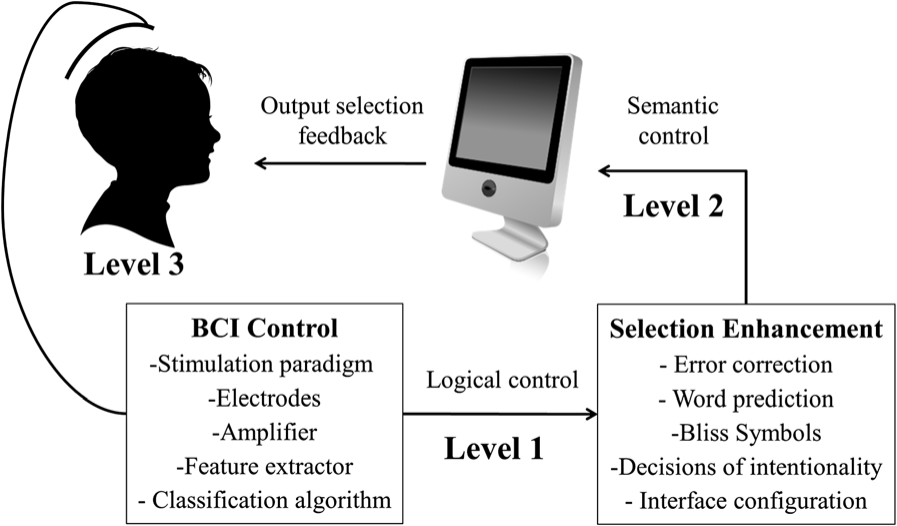
**Architecture of a BCI-based AAC system that is comprised of two modules: (1) a BCI Control Module that translates brain signals into logical control outputs and (2) a Selection Enhancement Module that translates logical control to semantic control.** Performance of BCI-based AAC systems can be measured at three levels (labeled Level 1, Level 2, Level 3) within this architecture; each level of measurement is currently assessed by a variety of often incommensurable performance metrics.

Augmentative and alternative communication (AAC) systems are used by individuals with communication disorders to supplement or replace speech or writing. A wide variety of AAC systems exist, ranging from picture and communication boards to speech generating devices [1,2]. At minimum, all AAC systems require the user to produce a binary signal to indicate voluntary selection of an output option. While many access technologies exist to translate residual motor functions into an output signal [3], those with the most severe motor disabilities do not have any voluntary muscle control and thus cannot access AAC technologies. For these individuals, brain-computer interface (BCI) technology can be used as a form of augmentative and alternative communication (AAC). The architecture of a BCI-based AAC system can be represented by many different frameworks [4]; herein, we model BCI-based AAC systems as two interconnected Modules, each of which is comprised of a number of functional components, depicted in Figure 1. The *BCI Control Module* translates a BCI user’s brain state into a logical control output. Its functional components may include a stimulus presentation paradigm which causes the BCI user to elicit particular brain states (e.g. the order of flashes in P300-based BCIs [5,6], stimulus configuration [7], stimulus colour [8], stimulus rate [9]); electrodes and amplifiers; feature extractors; and classification algorithms (e.g. co-adaptive calibration [10], adaptive online classification [11]). A comprehensive review of the variations of each of these functional components is provided in [12]. The BCI Control Module makes discrete selections from a system-dependent number of possible options. These selections are made independent of any semantic knowledge of the AAC interface, and the resulting logical control signal is sent to the Selection Enhancement Module. A *Selection Enhancement Module* translates this logical control to semantic control, using techniques ranging from direct association (e.g. one output option corresponds to one specific communicative symbol), to algorithms such as error correction and word prediction, to interface configuration (e.g. the Hex-o-Spell [13]). Selection enhancement is not unique to the BCI field – it is employed in auto-text correction such as T9 predictive text and Swype on mobile phones, automatic speech recognition and existing AAC interfaces. These two Modules work in tandem to provide a means of communication for individuals who have severe motor impairments that limit their ability to speak and to access traditional AAC devices.

Many variations of each of the components of both BCI Control and Selection Enhancement Modules exist, and can be combined together in multiple ways to produce unique BCI-based AAC system configurations. To develop an optimal BCI-based AAC technology, researchers must be able to compare each of these configurations to assess the relative benefit of each component to the overall communication capacity of the system. In other words, the quest for the best BCI requires efficient evaluation criteria for the performance of each component of the communication system.

As is the case with the evaluation of any AAC system, the issue of where to measure performance is paramount. There are three locations, or Levels, at which BCI-based AAC performance can be measured, as depicted in Figure 1. Level 1 performance is measured directly at the output of the BCI Control Module. Here, a logical control output without semantic meaning is generated. This output is a single selection of one output from a variable number of options presented on the user interface, such as one of six targets in the Hex-o-Spell BCI [13], or one of 36 options in a traditional P300 Speller [5]. Here, the effective generation of a logical control output is commonly assessed by measures of speed, accuracy, or a combination thereof, such as information transfer rate. To date, measurement of BCI performance has typically occurred at this Level. However, as BCI systems begin to explore improved user interfaces (e.g. integration of word prediction in spelling applications, innovative spelling systems, adaptive user interfaces [14,15]), Level 2 measures of communication capacity at the output of the Selection Enhancement Module have become more common [14-17]. Level 2 measures of BCI performance account for the fact that a single selection by the user may have different degrees of “power” in terms of what it can accomplish when the logical control signal has been interpreted by the Selection Enhancement Module. These two Levels mirror where performance has typically been measured in traditional AAC systems. Rate enhancement strategies such as the physical arrangement of a display, linguistic predictive capabilities, and cues such as colour and abbreviation expansion have been extensively explored by the AAC field to overcome the *rate problem* of the slow productions of augmentative communicators [18]. In the AAC field, Level 1 metrics include selections, switch activations, letters, words utterances, etc. per unit of time [19-22], and Level 2 metrics include metrics of text or selection savings [20-22], such as the Rate Index (average communication rate/selection rate) [23]. Finally, while the rarity of in-home BCIs being used by the target population have delayed the need to identify a higher-level measure of BCI performance, the AAC literature indicates that it is also possible to measure performance of a communication system, and therefore of a BCI, at the level of its impact on the user (e.g. [24,25]). This would be considered a Level 3 measurement of BCI performance, and can be assessed by determining whether the presence of a BCI leads to fuller, richer communication with a partner, or an improved quality of life.

A large number of performance metrics have been used in BCI research studies to quantify the communication capacity of a specific BCI system. While accuracy is typically reported, this metric has several major limitations, including not accounting for time and being biased by chance performance in systems with different numbers of output states. Consequently, research groups are developing and publishing their own custom performance metrics. For example, in order to report the performance of a BCI where users were given the option of correcting mistakes that they had made in typing a sentence, Townsend et al. [6] developed the “practical bit rate” while Jin et al. [7] used the practical bit rate, with the addition of the “written symbol rate”.

To determine the variety of metrics in use, we conducted a literature review in Web of Science, combining the keywords “*brain-computer interface*” and “*communication*”. The search was limited to English communications in peer-reviewed journals dating between January 2005 and January 2012. Articles were included if they described the performance of synchronous, “goal selection” BCIs [26] used by human participants for communication. According to these criteria, 72 articles were retained and included in the appraisal.

Within these 72 articles, 12 different combinations of metrics were used to describe BCI-based AAC performance. These combinations and their frequency of use are listed in Table 1.

**Table 1:** Metrics used in the literature from January 2005 – January 2012 to report performance of communication-based BCIs

**Metrics reported**	**Number of times reported**	**Article references**
Accuracy	38	[8,27-63]
Accuracy and information transfer rate (ITR)	16	[6,7,64-77]
Information transfer rate (ITR)	7	[78-84]
True and false positives	1	[85]
Accuracy and written symbol rate (WSR)	1	[86]
Accuracy and speed	1	[9]
Accuracy and mutual information	1	[11]
Accuracy and number of errors	1	[87]
Accuracy and selections per minute	1	[88]
Accuracy, bit rate, selections per minute, output characters per minute	1	[14]
Characters per minute	1	[89]
Accuracy, information transfer rate (ITR), NASA task load index, QUEST 2.0	1	[90]

The scope and type of metrics that have been used in the BCI field over the 7 years reviewed by the authors present two major problems. First, many of the metrics are incommensurate, precluding the comparison of results between different BCI studies. This presents a serious limitation to the growth and development that is possible in the BCI field. Second, many metrics are based on digital communication theory, and thus on assumptions that do not necessarily hold for BCI-based communication. In digital communication, a large quantity of data is typically available in order to measure performance. Due to the relatively slow communication speed of BCIs and human factors such as fatigue, it is rare that large amounts of data are available to quantify BCI performance. For example, within the P300-based BCI studies included in the surveyed literature, measures of performance were derived from an average of 30 selections, while the average P300 BCI had 36 classes. Consequently, many of the metrics that are used as gold standards in digital communication cannot necessarily be applied to the BCI field, resulting in the need for new field-specific standards to be developed.

As BCI-based AAC research continues to grow in popularity, there is a pressing need for the acceptance of standardized BCI evaluation metrics that can be used to report performance in any study using a BCI for AAC. Such metrics would enable the efficient comparison of various BCI components, accelerating the development of a practical, efficient BCI that can be used by individuals with severe motor impairments for the purposes of communication. This manuscript will compare the performance metrics that have been used for Level 1 and Level 2 measurement of BCI-based AAC performance, and recommend a standard metric for each level. Level 3 metrics will not be addressed; the interested reader is referred to the literature regarding measuring assistive technology impact on the user [91-93].

## Level 1 performance metrics

### Types of BCI control modules

BCI Control Modules for AAC utilize pattern recognition techniques to translate the electrical signals generated from the brain states of BCI users into the selection of one discrete option from a list of available outputs. The BCI Control Module functions under the assumption that (1) specific mental operations or (2) responses to specific sensory stimuli result in reproducible frequency or event-related potential patterns. Thus, two types of BCI Control Modules can be distinguished: (1) endogenous control modules, which respond to spontaneous control signals from the user (e.g. motor imagery to generate sensorimotor rhythms (SMRs)), and (2) exogenous control modules, which respond to control signals evoked from the user by a stimulus (e.g. event-related potentials such as the P300 response, or visually-evoked potentials (VEPs)) [12]. Effective Level 1 performance metrics should enable comparison within and between both types of BCI Control Modules.

### Evaluation criteria for level 1 performance metrics

We define four criteria (described in detail below) for the evaluation of common Level 1 performance metrics. An effective Level 1 metric would be able to capture the performance of a maximal number of BCI-based AAC systems. While future systems may be created that cannot be measured with existing metrics, we offer the following criteria: the metric should have the ability to capture (1) throughput (*throughput*), (2) the performance of a BCI with a variable number of categorical outputs (*categorical outputs)*, and (3) unbiased performance (*unbiased)*. Furthermore, the metric should (4) be practically communicable to researchers and clinicians from various disciplines working in the field of BCI and practically calculable from the amounts of data typically gathered in BCI experiments (*practicality*).

#### Throughput

BCI Control Modules must balance a tradeoff between system speed and system accuracy. While accuracy is commonly reported, the time per decision varies widely between different BCIs. In offline analysis, this parameter is often artificially varied to investigate the ideal settings for a system, so that the time per decision may not be fixed even within a given study. Effective Level 1 metrics must therefore capture system throughput (information per time). Metrics that report throughput can allow direct comparisons of varied BCI types; such metrics also allow comparisons between different configurations of the same BCI, such as those used to optimize parameter settings.

#### Categorical outputs

In any BCI system, output may be discrete or continuous; equivalently, in BCI-based AAC systems output may be (1) categorical (e.g. letters from the alphabet in a P300 speller) or (2) ordinal targets (e.g. targets in a one-dimensional SMR-based BCI). User performance in selecting ordinal targets is often measured by metrics that require inter-target distances as input (e.g. mean squared error); these metrics are thus not compatible with categorical data. By contrast, metrics designed for categorical outputs can be used with ordinal outputs, though they will ignore the extra information gained from the labels. Thus, Level 1 metrics must support categorical outputs to allow comparison between varied BCI types.

#### Unbiased

The reported performance of BCI Control Modules can be biased by three factors. 1) A variable number of discrete outcomes. P300-based BCI spellers enable the user to select from many options within a single trial (e.g. 4 options [94], 36 options [5], and 72 options [6]), whereas some mu-rhythm based BCI selection tasks only permit selection between two discrete outputs within a single trial [95]. Chance performance of the BCI Control Module is inversely related to the number of options. 2) Experimental bias, the marginal distribution of the intended BCI outputs determined by the experiment. In other words, the potential bias that is introduced if a BCI user is instructed to select one output option more frequently than others, as is likely to be the case during communication even outside of the laboratory. 3) Classifier bias, where a BCI Control Module preferentially selects one class over others. To enable efficient comparison between different BCI Control Modules, Level 1 metrics must be unbiased by any of these factors.

#### Practicality

An effective metric must be practicably communicable between various research groups and accessible to individuals from various disciplinary backgrounds working in the BCI field. The metric must present BCI performance in a form that is practical for journal articles and sufficiently simple to be understood by those without engineering expertise. The metric must also be practically calculable from the relatively small amounts of data that are gathered from AAC-based BCI experiments with human participants. This final point is particularly significant, as communication systems such as spellers tend to have a large number of output options (classes), leading to a small number of examples per class in a typical experiment.

### Common level 1 performance metrics

In light of the four criteria defined above, we present and discuss five common Level 1 performance metrics in this section: (1) error rate or classification accuracy; (2) Cohen’s Kappa coefficient; (3) confusion matrix; (4) mutual information; and (5) information transfer rate or bit rate. The discussion is summarized in Table 2. It is possible to address the limitations of some metrics through relatively minor adjustments (e.g. in addition to accuracy, one can report time per sequence and time between characters in a P300-speller BCI to enable the derivation of ITR). However, as these metrics are often reported without the information necessary for such conversions, they will be evaluated according to the four criteria under the assumption that no further information about performance is provided. Several other Level 1 BCI performance metrics exist that are typically used to measure continuous BCI output, such as the correlation coefficient and mean square error [96]. These metrics are often used in SMR-based BCIs, but as they cannot be used with the categorical output generated by some BCI-based AAC systems, they will not be discussed further in this paper.

(1)  Error rate or classification accuracy [98]

 This metric determines how often the BCI makes a correct selection; in other words, the percentage of total selections that are correct. While it is the most intuitive metric of BCI performance, it does not account for time, often suggesting that BCI performance increases monotonically with time per decision. Furthermore, this metric is biased by the chance performance of AAC configurations with different numbers of discrete outcomes, and assumes the existence of a single accuracy which is uniform across all possible outputs.

(2)  Cohen’s Kappa coefficient [99,100]

 Cohen’s Kappa is a measure of the agreement of two observers; for a BCI-based AAC system, it is used as a measure of agreement between the correct output and the BCI Control Module output. Like classification accuracy, this metric does not account for the time required to make a selection, and does not give a measure of throughput. While Cohen’s Kappa is designed to account for chance agreement, it can produce unexpected results if the underlying class distribution is biased [101].

(3)  Confusion matrix [102]

 For BCI-based AAC systems, a confusion matrix is a matrix with correct output as rows, BCI Control Module outputs as columns, and the number of occurrences in the intersections. The diagonal therefore represents the number of correct outputs. The confusion matrix does not account for time and thus does not measure throughput. The relative sums of the rows of the matrix reveals the frequency of each intended output in the experiment, thus, confusion matrices can show patterns in the error distribution (e.g. row-column errors in the P300 Speller).

 The confusion matrix is often not practically calculable. Every entry in the matrix is proportional to a probability density estimate for a particular combination of correct and actual outputs; the number of density estimates that are required thus grows as the square of the number of states. Particularly in P300 experiments where 36 or more possible outputs are typical, this amount of data is rarely available. Also, this metric is often not practically communicable. While confusion matrices are 2-D when representing the performance of one specific BCI configuration; representing the performance across a varying number of stimulus presentations requires reporting a 3-D matrix. In addition, while the 2-D matrices are easily reported for BCIs with a small number of total possible outputs, they become impractically large for BCIs with a large number of total possible outputs. The classical Farwell and Donchin 36-class P300 speller would *a priori* require reporting a matrix with 1296 entries [5]. Since that particular implementation makes separate decisions for rows and columns, reporting two separate, 36-element confusion matrices may be sufficient, though this requires an assumption of independence and makes interpretation more difficult. Some modern spellers, such as Townsend’s checkerboard speller [6], do not share this structure and would thus require reporting the full N^2^ entries (5184 for the Townsend speller). Furthermore, most of the entries are small in value, and therefore difficult to measure accurately. The combination of these factors makes the confusion matrix impractical.

(4)  Mutual information [103,104] or Nykopp’s bit rate [97]

 Mutual information is a measure of the overlap between the correct output and the output of the BCI Control Module; it is a measure, in bits, of the throughput of information from the BCI. Since its formula includes marginal and error probabilities, it is robust with respect to experimental and system bias. However, to account for these sources of bias, the calculation of mutual information requires the estimation of the joint statistical distribution of the input and output; the amount of information needed for this estimation scales as the square of the number of total possible outputs of the system, making it impractical for use in a realistic setting with a BCI with a high number of possible outputs, such as a P300 Speller.

 Nykopp’s bit rate is equivalent to mutual information, though this metric does not require the explicit estimation of joint statistical distribution. While Nykopp recommended maximizing the throughput measure by taking the maximum mutual information across all marginal input probabilities, this is an artificial approach, and the metric is only valid provided this maximization is not performed [105].

(5)  Wolpaw’s Information transfer rate (ITR) or bit rate [104].

 Information transfer rate (ITR), also called bit rate, measures the amount of information passing through a device per unit time. It is derived from mutual information, thus, it works with categorical outputs. It is worthwhile to note that in the derivation, Wolpaw et al. [104] assumed that the probability of error would be uniform across all possible outputs, and that errors would be uniformly distributed among the available choices. The violation of these assumptions can produce unexpected results, as shown in [105]. However, these assumptions dramatically limit the amount of data necessary to calculate the metric.

**Table 2:** Comparison of common Level 1 BCI-based AAC performance metrics

**Metric**	**Description/Equation**	**Evaluation criteria**			
		**Throughput**	**Categorical outputs**	**Unbiased**	**Practicality**
Accuracy/Error Rate	*Acc* = *P*; *Err* = (1 - *P*)		✓		✓
Cohen’s Kappa	$\kappa =\frac{P-1/N}{1-1/N}$		✓		✓
Confusion Matrix	A matrix with intended (true) outputs as rows, actual outputs as columns, and the number of occurrences in the intersections.		✓	✓	
IMutual Information or the formulation in [97]	$I\left(X;Y\right)=\sum_{Y}\sum_{X}p\left(x,y\right)\times \log\left(\frac{p\left(x,y\right)}{p\left(x\right)*p\left(y\right)}\right)$ (Note this can be used to measure throughput rate by simply dividing by the time per trial)	✓	✓	✓	
Information Transfer Rate (ITR)	$\mathrm{ITR}=\frac{1}{c}\left(\log_{2}\left(N\right)+P\log_{2}\left(P\right)+\left(1-P\right)\log_{2}\left(\frac{1-P}{N-1}\right)\right)$	✓	✓		✓

**P**: probability of correct selection; **N**: number of choices; **p(x)**: marginal distribution of X; **p(x,y)**: joint distribution of X and Y; **c:** time per selection.

Check marks indicate that the metric fulfills the evaluation criterion.

### Level 1 performance metric recommendations

As illustrated in Table 2, none of the metrics that are currently used to report performance of a BCI Control Module satisfy all four criteria of an effective Level 1 metric. Mutual information and information transfer rate both satisfy three of the four evaluation criteria. Each has a different strength: mutual information accounts for bias, while information transfer rate is more practical to calculate in light of typical data limitations of BCI experiments. Fortunately, as ITR was derived from mutual information, they are commensurable metrics. Thus, the decision of which of these two metrics to use can be guided by data availability. Standard formulas can be used to calculate the confidence bounds of each class, since the accuracy of each class is observed as a binomial random variable. If sufficient data are available to demonstrate a significant difference between classes, mutual information is preferred. However, if sufficient data are not available, the error from using poor per-class estimates of accuracy may be worse than the error expected from assuming a single uniform accuracy. We therefore recommend the use of **mutual information** when significant bias is expected or deliberately introduced, and the **ITR** approximation in other situations, as the standard metrics to report Level 1 performance of any BCI Control Modules.

## Level 2 performance metrics

### Types of selection enhancement modules

Three types of Selection Enhancement Modules can be defined based on their respective mechanisms for enhancing the logical output they receive from the BCI Control Module: (1) automatic error correction; (2) rate enhancement; and (3) control state detection.

1. Automatic error correction mechanisms are ways by which the system can recover from errors. These mechanisms include techniques such as the using the detection of an “error potential” brainwave to automatically undo erroneous selections [106], or the replacement of a deleted character with a likely second candidate upon the selection of a backspace [17].

2. Rate enhancement mechanisms map the discrete logical selection received from the BCI Control Module into selections with a larger unit of semantic meaning. Such mechanisms range from populating selection options with communicative signs and symbols, e.g. Blissymbols [107], to enhancing a P300 speller with word prediction, which enables users to complete full words in a single selection (e.g. [14]).

3. Control state detection mechanisms monitor the attention of the user, and abstain from making a selection when the user is not paying attention to the BCI, thus preventing selections which are likely to be erroneous [108].

While all three mechanisms operate on different principles, they are each designed with the common purpose of enhancing the effectiveness of BCI-based communication beyond what is possible with a BCI Control Module alone.

### Evaluation criteria for level 2 performance metrics

In order to enable BCI researchers and users to make informed choices of the best Selection Enhancement Modules, it is imperative to have a metric that allows comparison within and across all three Selection Enhancement mechanisms described in section “Types of Selection Enhancement Modules”. In addition, the metric should be usable with different subject instructions regarding the handling of errors specified in an experimental protocol (e.g. user required to correct errors, user required to ignore errors). Finally, the criterion of practicality as defined for Level 1 metrics also apply for Level 2 performance metrics. The practicality criterion is even more important for Level 2 metrics than for Level 1 metrics, as it is likely that a broader range of disciplines will be interested in Level 2 measures of performance. Thus, a Level 2 metric must be compatible with the three types of Selection Enhancement mechanisms - 1) automatic error correction, 2) rate enhancement and 3) control state detection. It should also be compatible with 4) experimental protocols with and without error correction by the user; and should 5) be practicality calculable and communicable.

### Common level 2 performance metrics

In the current BCI literature, six Level 2 metrics are used: (1) the written symbol rate, (2) practical bit rate (3) the extended confusion matrix, (4) the system efficiency, denoted - “Eff_sys_”, (5) output characters per minute and (6) the BCI-Utility metric. We describe and discuss these metrics with respect to the five criteria presented in section “Evaluation criteria for level 2 performance metrics”. The results of the comparison are presented in Table 3.

(1)  Written symbol rate [15]

Written symbol rate (WSR) is primarily applicable to error correction mechanisms in Selection Enhancement Modules. The formula accounts for the cost of selecting an erroneous character – selecting a backspace, then selecting the correct character – and for the fact that each selection involved in correcting the error is subject to error itself. However, the WSR strictly underestimates system performance, especially for low accuracies, as the formula uses ITR to derive the symbol rate. ITR already includes theoretical error correction; thus WSR accounts for each error twice, making it an invalid measure.

(2)  Practical bit rate [6]

Like the WSR, practical bit rate is primarily applicable to error correction mechanisms in Selection Enhancement Modules. To accurately represent real-world communication scenarios, the formula adds a penalty of two additional selections for every error incurred, accounting for the same likelihood of making an error during the correcting process as in the original attempt. The formula used to calculate this metric is the same as that used for the BCI-Utility metric in the case where error correction is performed through a backspace entry in the matrix, but is less flexible in that no alternative form exists for other scenarios.

(3)  Extended confusion matrix [17]

The extended confusion matrix (ECM) is an extension of the confusion matrix (described in section “Common level 1 performance metrics”) that accounts for abstentions, or situations where the BCI system deliberately decides not to output a selection. However, it requires the collection of sufficient data to provide estimates of each probability of misclassification. Thus, like mutual information or confusion matrices among the Level 1 metrics, ECM requires more information than is available in many BCI experiments (e.g. ECMs for spellers could require thousands of entries, and at best are impractical to both report and interpret).

The problem of impracticality could be reduced by reporting aggregate data from all subjects; however, this approach introduces subtle biases into the data. As examples: the backspace option is more likely to be selected by individuals with poor performance; in time-limited trials, only participants with good performance will complete the sentence, thus characters appearing earlier in the sentence are likely to show a bias towards poor performance. These subtle factors mean that even aggregate data must be reported and interpreted with caution.

ECM also does not currently have an explicit mechanism for capturing selection enhancements such as word prediction or symbolic communication. It may be possible to derive an extension that could capture these enhancements, however such a derivation is likely to require considerable effort.

(4)  Eff_sys_[17]

 Eff_sys_ is a measure of the efficiency of a BCI system. Eff_sys_ is based on ECM, but differs in that it (a) includes calculations for the cost of errors; and (b) is a scalar metric, and therefore practical for publication. Eff_sys_ is designed to account for the fact that different outputs may have different probabilities of correct classification; however, its derivation assumes that the probability of selecting a ‘backspace’ option is equal to the probability of selecting each of the other outputs. This inconsistency leads to erratic behavior in this metric; if the accuracy of even one potential output is less than 50%, Eff_sys_ = 0. This behavior can be corrected by a slight modification to the formula, which we present as Eff_sys_’.

(5)  Eff_sys_’

 Eff_sys_’ is a modification of Eff_sys_ that accounts for the fact that different outputs may have different probabilities of correct classification. The formula presented in Table 3 is derived for the conditions of: (1) a BCI user selecting outputs (e.g. letters) with the option of undoing erroneous selections; and (2) an erroneous selection requiring the selection of an ‘undo’ option, followed by reselecting the intended output. The second condition is not true in the case of an erroneous selection of the ‘undo’ option; consequently, Eff_sys_’ will slightly underestimate BCI performance. This formula allows all outputs except the ‘undo’ option to have any non-zero probability; only the ‘undo’ option is required to have accuracy greater than 50%. Note that this form of the metric does not account for Selection Enhancement Modules that implement automatic error correction, though it approximates user-based error correction better than Eff_sys_.

(6)  Output characters per minute [14]

 Output characters per minute (OCM) is calculated by dividing the final length of the error-corrected output by the time required to accomplish the task. The metric has the ability to capture the performance of all three types of Selection Enhancement Modules. However, as currently presented, the metric is not applicable to experimental protocols where errors remain in the final text, and it unduly penalizes system performance in situations where users did not notice an error immediately and continued typing before returning to correct the mistake. Furthermore, OCM is restricted to character-based communication. Although the majority of BCI research has focused on spelling applications, there are a wide variety of AAC systems that take advantage of symbolic or pictorial communication. To improve communication efficiency, BCI-based AAC systems will likely adapt these well-established conventions from the AAC field; the performance of such BCI systems is impossible to capture with OCM.

(7)  BCI-Utility metric [16]

 The BCI-Utility metric is the ratio of the expected benefit per selection and the expected time per selection. The expected benefit may be greater than one, as in the case of Selection Enhancement Modules such as word-prediction; equal to one, as in the case of direct association of a single selection to a single letter; or less than one, as in the case of the Hex-o-Spell BCI [13] where the selection of a single hex is one of two selections necessary to generate an output letter. BCI-Utility is able to effectively measure the performance of all three types of Selection Enhancement Modules, and is applicable to experimental protocols with and without error correction. Dal Seno et al. [16] present several forms of the metric; in Table 3, we present the most general form of the metric. If none of the presented forms are appropriate, researchers may derive a new form from its basic principles to account for the specific implementation of their BCI-based AAC system. A simple example would be adding a modification for non-uniform accuracy across all possible outputs. Consequently, the BCI-Utility metric can be extended to performance enhancements that its creators did not anticipate. Indeed, the BCI-Utility metric may also be appropriate for BCIs designed for purposes other than communication. Unfortunately, as standard formulas are not available for several common scenarios, there is some possibility that errors in derivation by research groups could lead to conflicting results. While this could be addressed by a set of standard guidelines for the use of the metric, these do not currently exist; thus, the practicality of calculating the metric is not ideal at present.

**Table 3:** Comparison of common Level 2 BCI-Based AAC performance metrics

**Metric**	**Description/Equation**	**Evaluation criteria**				
		**Error correction**	**Rate enhancement**	**Control State detection**	**Experimental protocol**	**Practicality**
Written symbol rate (WSR)	$\mathrm{WSR}=\left\{\begin{array}{c} \frac{\left(2\mathrm{SR}-1\right)}{c}\\ 0 \end{array}\right)\begin{array}{c} ,\mathrm{SR}>0.5\\ ,\mathrm{else} \end{array}$ where *SR* = *ITR* × *c*/**log**_2_(*N*)					✓
Practical bit rate (PBR)	$\mathrm{PBR}=\left\{\begin{array}{c} \frac{\left(2P-1\right)}{c}\\ 0 \end{array}\right)\times \log_{2}\left(N\right)\begin{array}{c} ,P>0.5\\ ,\mathrm{else} \end{array}$ The original reference did not report a formula, this was back-calculated from the results				✓	✓
Extended confusion matrix (ECM)	Confusion matrix, as in Table 2, extended to allow for an extra detected class, “abstention.”	✓		✓	✓	
Eff_SYS_	$\mathrm{Ef}f_{\mathrm{SYS}}=\frac{1}{\bar{L_{\mathrm{CW}}}\times \bar{\mathrm{ESC}}}$ Where $\bar{\mathrm{ESC}}=\sum_{i=1}^{N_{\mathrm{LA}}}\frac{\hat{p}\left(i\right)}{2p\left(i\right)-1}$ Where L_CW_ is the mean codeword length, N_LA_ is the number of symbols in the logical alphabet, $\hat{p}$ is the probability of a logical output appearing, and p(i) is the probability of identifying output i. This equation is simplified from equation (20) of [17], assuming that all errors require two selections to correct. Interested readers are referred to [17] for alternative methods of error correction.	✓		✓		✓
Eff_SYS_’	EffSYS'=1LCW¯×ESC¯ Where $\mathrm{ESC}=\sum_{i=1}^{N_{\mathrm{LA}}}\hat{p}\times \left(\frac{1}{p\left(i\right)}+\left(\frac{1}{p\left(i\right)}-1\right)\left(\frac{1}{2*p_{B}-1}\right)\right)$ Where L_CW_ is the mean codeword length, N_LA_ is the number of symbols in the logical alphabet, and $\hat{p}$ is the probability of a logical output appearing, as per [6]. The symbols p(i) and p_b_ are the probabilities of correctly identifying output i and the backspace option, respectively.			✓	✓	✓
Output characters per minute (OCM)	$\mathrm{OCM}=\frac{\mathrm{Correct}\;\mathrm{Characters}}{\mathrm{Time}\;\mathrm{Taken}}$	✓	✓	✓		✓
BCI-Utility metric	$U=\frac{E\left(\mathrm{benefit}/\mathrm{selection}\right)}{E\left(\mathrm{time}/\mathrm{selection}\right)}$ *U* must be derived for particular problems and can take different forms, several of which are presented in [16]. Several of these forms are redundant with other level 2 metrics, for example, in the experiment presented in [16], this metric becomes identical to practical bit rate.	✓	✓	✓	✓	*

c: time per selection; N: number of choices; ITR: information transfer rate; P: probability of correct selection.

*: Not currently practical, but can be with future work.

Check marks indicate that the metric fulfills the evaluation criterion.

### Comparison of level 2 performance metrics

**Figure 2:**
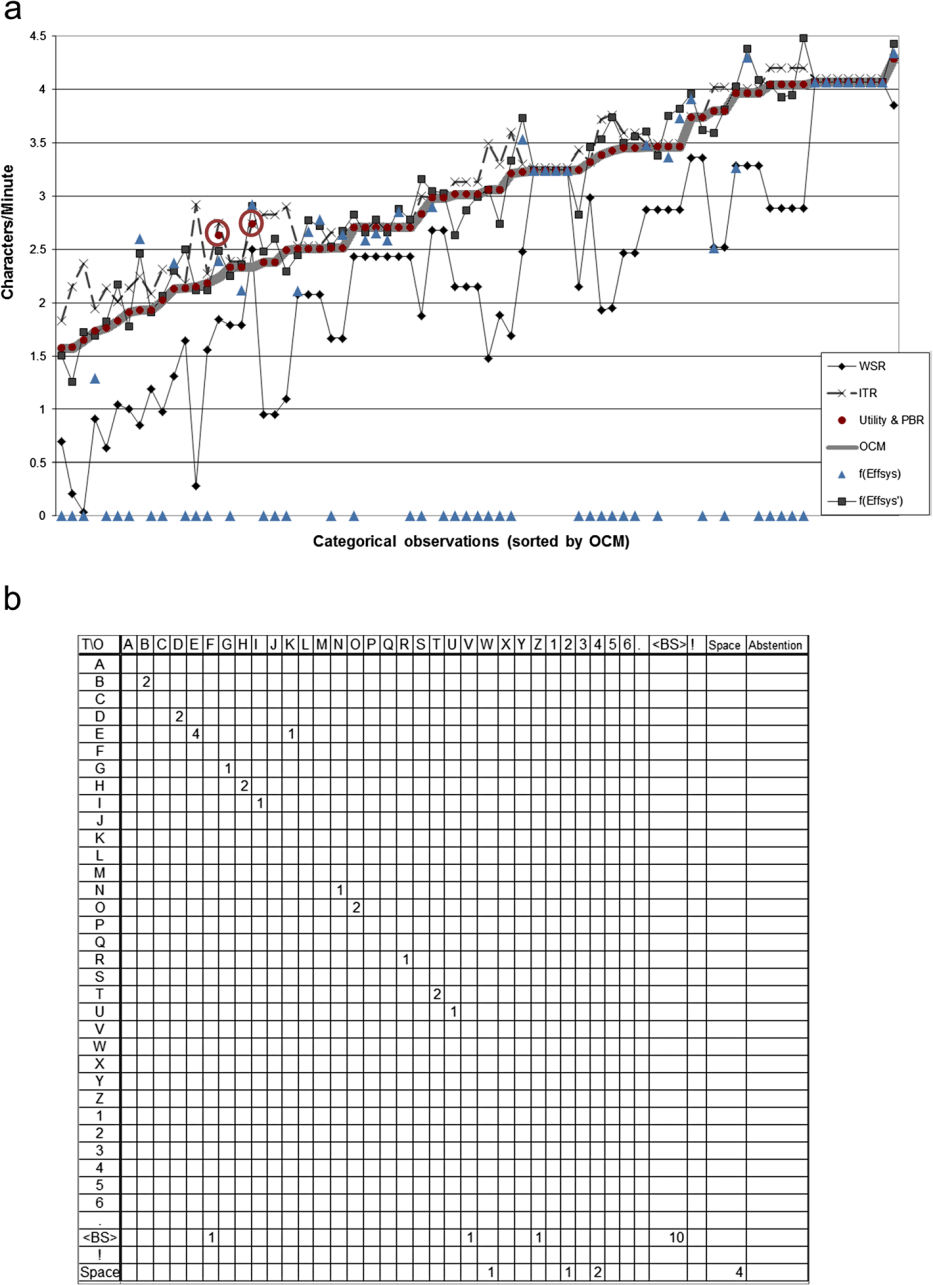
**Comparison of performance of Level 2 metrics. a**) Comparison of each scalar Level 2 metric on data from a P300 copy-spelling task with correction, 75 sentences from 22 users, sorted by OCM. All metrics were converted into characters per minute (e.g. f(Eff_sys_) and f(Eff_sys_’) represent the metric multiplied by the character output rate). **b**) The ECM for the first observation presented in a), in a format required by checkerboard-style spellers. Note the sparsity of the matrix, even though data from fifteen minutes of BCI use are included. For practicality, the 74 ECMs corresponding to the other observations are not presented.

To further illustrate the differences between these seven Level 2 metrics, a comparison is provided in Figure 2. Data was collected in the 3-session experiment performed in [109]. Briefly, participants (n = 22, including 9 with amyotrophic lateral sclerosis) were asked to copy a total of 9 sentences, each 23 characters in length, using a classic P300 Speller BCI [5]. Stimuli were presented with a 31.25 ms flash, an inter-flash interval of 125 ms, and 3.5 seconds between characters. A least-squares classifier was used to determine the logical control output of this BCI Control Module. A simple direct-association Selection Enhancement Module mapped the logical control output to a single alphanumeric character, which was displayed on the computer screen. Participants corrected errors using a backspace option in the BCI. Sentences were excluded if the participant did not complete the full sentence, correcting all errors, within 15 minutes. The 75 sentences with the lowest OCM are reported in Figure 2.

In this dataset, OCM and BCI-Utility differed only on two data points (circled). In both cases, the user noticed an error only after typing several correct letters, and had to erase those letters to correct the mistake. Thus, the BCI-Utility represents an estimate of the OCM the system would have achieved without user error. WSR severely underestimates performance, while ITR overestimates performance (particularly for low accuracies), and Eff_sys_ estimates the performance of 40 of the sentences to be zero. Both Eff_sys_ and Eff_sys_’ vary around OCM; this behavior demonstrates that for small datasets, the error from assuming uniform accuracy across classes is smaller than the measurement error on individual accuracy estimates. The ECM for the first observation of Figure 2a is presented in Figure 2b; the size and the sparsity of the matrix for a single observation illustrates the lack of practicality of using this metric to report BCI-based AAC performance.

### Level 2 performance metric recommendations

As illustrated in Table 3, none of the metrics that are currently used to report performance of a BCI Selection Enhancement Module satisfy all five criteria of an effective Level 2 metric. The BCI Utility-metric and OCM both satisfy four of the five evaluation criteria. OCM has several limitations: it cannot be calculated if errors remain in the text, thus, it is incompatible with a large number of experiments that do not require user-driven error correction; it is also unable to measure performance from symbolic communication. The BCI-Utility metric is compatible with all types of Selection Enhancement Modules and experimental protocols, but has limited practicality in that it does not have a fixed form and needs to be derived for particular problems. This limitation is easily addressable through future development of standards that delineate specific formulas and guidelines for the BCI-Utility metric under varying experimental paradigms. Thus, we recommend the use of the BCI-Utility metric as the standard to report Level 2 performance of any BCI, enabling the efficient comparison of Selection Enhancement Modules. Level 1 metrics should also be reported in any research involving Selection Enhancement, so that the effect of the Selection Enhancement Module can be clearly seen, and underlying experimental differences due to different BCI Control Modules can be identified.

## Discussion

### Recommendations

**Figure 3:**
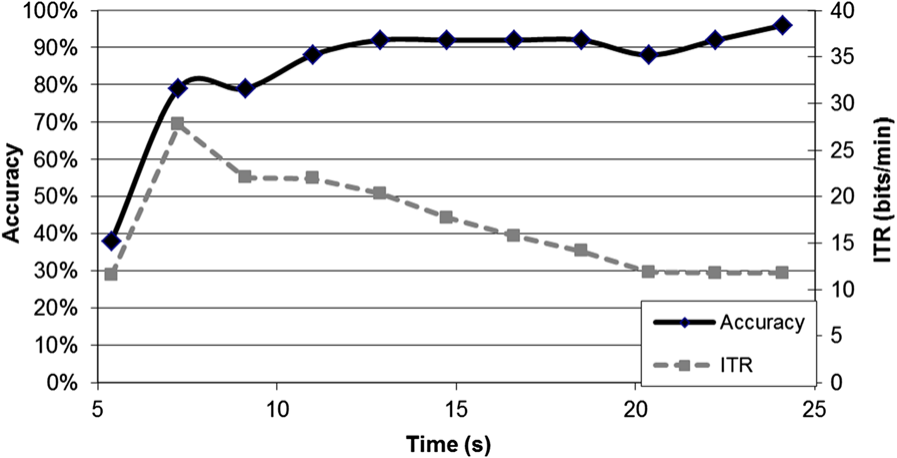
**Example of augmented level 1 performance metric for a P300-speller BCI.** Both the ITR and the accuracy are reported with respect to time, enabling comparison with other BCI Control Modules. Note that ITR was calculated including the time between selections.

The continued popularity of research in and development of BCIs has created a pressing need for the adoption of standardized BCI evaluation metrics that can be used to report performance for BCI-based AAC systems. Without such metrics, BCI studies that demonstrate the performance of various BCI Control Module or Selection Enhancement Module components remain incommensurable, preventing comparisons of BCI function between labs. This severely limits progress toward developing a practical, efficient BCI that can be used for communication by individuals with severe motor impairments. Based on criteria chosen to maximize comparability between all variations of BCI-based AAC systems, we make the following recommendations:

1. Using **mutual information/information transfer rate (ITR)** as the standard metric for reporting Level 1 BCI performance, and the **BCI-Utility metric** as the standard metric for reporting Level 2 BCI performance.

2. Supplementing these standard metrics with specific metrics typically used for a particular BCI paradigm. For example, in the P300-Speller BCI, the accuracy of the system versus the number of stimulus presentations is typically reported; in this situation, we recommend reporting accuracy versus time, with ITR overlaid on top, as presented in Figure 3. This figure is commonly used for selecting speed of operation, and would not be possible without a practical metric for measuring throughput. Note that BCI-Utility should also be reported. Such a graph is not applicable for endogenous BCIs such as those controlled by SMRs, where the BCI user is presented with constant feedback; for these systems, reporting accuracy, ITR, and BCI-Utility of the system using online settings is sufficient.

3. Reporting both Level 1 and Level 2 metrics in all BCI studies, but particularly Selection Enhancement Module studies. The performance of BCI systems with Selection Enhancement Modules is dependent upon the performance of the BCI Control Module as well as the performance of the Selection Enhancement Module. Reporting both metrics enables the performance of each module to be assessed independently. Similarly, when BCI systems are eventually assessed at the level of the user, it will be important to report Level 1, Level 2 and Level 3 metrics simultaneously, so that effective comparisons can be drawn between different BCI systems.

### Limitations

While the recommended metrics enable efficient comparison of most existing BCI systems, they may be limited in their ability to measure the performance of BCI systems that are developed under control paradigms other than those mentioned in this paper. For example, theoretical self-paced BCIs are continuously available to the user, and aware of when the user is engaging with the BCI interface or paying attention to something else (i.e. they support no-control states) [110,111]. Neither of the recommended metrics would be adequate to measure performance of such a system. Further, throughput is not necessarily a key metric for self-paced BCIs, which incorporate potentially long periods of subject inaction. Depending on the application, other metrics may be more suitable. For example, in applying a BCI to operate a call bell in a hospital environment, a metric such as precision-recall curves [112] or receiver-operator characteristics (ROC) [113] may be appropriate [66]. However, if self-paced BCIs are used to communicate frequently, this would likely be accomplished through a scanning system where options were selected via a single switch (e.g. [114]). In this case, the BCI-Utility metric could be extended through careful measurement of the average time per selection and accuracy of selection achieved.

The recommendations in section “Recommendations” are specific to BCIs used for AAC, which are goal selection BCIs. Process control BCIs, such as those used for the purposes of mobility or environmental control (e.g. to drive a power wheelchair or to operate a call-button) may use different evaluation criteria to select efficient performance metrics, as system accuracy is often more important than throughput. In such situations, the benefit of each selection (a critical concept in BCI-Utility) may be difficult to define. Therefore, while we recommend reporting the above metrics in any BCI research that includes communication, we do not expect the metrics to capture all aspects of system performance outside the realm of BCI-based AAC systems.

The selection of standard metrics to report Level 1 and Level 2 BCI-based AAC performances is a critical first step in enabling effective comparison of various BCI systems used for communication. The adoption of these metrics as the standard in the field is necessary, but not sufficient, to achieve this goal. A set of guidelines must also be established within the BCI field that detail the appropriate ways of presenting and using each of the metrics recommended in this review. Examples of issues to be resolved in future guidelines are: ITR has sometimes been reported with the pause between characters in the P300 Speller removed, which makes comparison between studies difficult; BCI-Utility metric is only effective when comparing symbol-based versus letter-based selections if the relative benefit of symbolic-based communication is provided. This review provides a foundation for the development of such guidelines; future work in this direction is encouraged in order to develop widely-accepted standards that are used to report BCI-based AAC performance using these recommended metrics. Of particular importance is the development of guidelines for use of the BCI-Utility metric and example derivations that extend those presented in [16]. While this is the only level 2 metric capable of capturing and comparing all of the Selection Enhancement research currently being conducted with BCIs, it currently runs the risk of leading to confusion or misinterpretation if different definitions of the “benefit” of a selection are used. Finally, as BCIs transition from laboratory-based technologies to home-based technologies, the development of standard Level 3 metrics will be necessary to facilitate the comparison and development of effective BCI-based AAC systems that can be used by individuals with severe motor impairments in a naturalistic communication setting.

Finally, it is important to recognize that in spite of our best efforts, there are experimental factors that potentially bias comparisons that cannot be corrected for by any single metric. Information about performance is always obtained under a restricted set of parameters that may favor one device over another. Standardizing the metrics used by the BCI field is advantageous to all involved, however, researchers must be vigilant against the biases inherent in each metric to ensure fair comparison of the performance of different BCI systems.

## Conclusion

Based on the criteria proposed in this paper, we recommend that when results of BCI-based AAC studies are disseminated: (1) Mutual Information or ITR should be used to report Level 1 BCI performance, depending on the amount of data available and the presence of bias, and the BCI-Utility metric should be used to report Level 2 BCI performance; (2) these metrics should be supplemented by information specific to each unique BCI configuration (see Figure 3 as an example); and (3) studies involving Selection Enhancement Modules should report performance at both Level 1 and Level 2 in the BCI system. Following these recommendations will enable efficient comparison between both BCI Control and Selection Enhancement Modules, accelerating the development of a practical, efficient BCI that can be used by individuals with severe motor impairments for the purposes of communication.

## Competing interests

The authors declare that they have no competing interests.

## Authors’ contributions

DET performed the mathematical analysis, summarized and rated each metric. He also developed the criteria jointly with SBM. SBM performed the literature review, developed the conceptual framework, and prepared the manuscript. JEH participated in the discussion of metrics, helping to shape the selection criteria and select metrics for detailed analysis. All authors read and approved the final manuscript.
